# Developing a framework for cash transfer programs that foster sustained economic empowerment to reduce sexual risk among adolescent girls and young women: a qualitative study

**DOI:** 10.1186/s12889-020-10130-8

**Published:** 2021-01-11

**Authors:** Alok Gangaramany, Peter Balvanz, Margaret Waruguru Gichane, Stephan Goetschius, Saransh Sharma, Krittika Sharma, Jeff Mulhausen, Paul Noble-Campbell, Joyce Wamoyi, Suzanne Maman, Ram Prasad

**Affiliations:** 1 Final Mile Consulting LLC, 141 W. Jackson Blvd, Suite 3302, Chicago, IL 60604 USA; 2 Department of Health Behavior, Gillings School of Global Public Health, University of North Carolina at Chapel Hill, 135 Dauer Dr, Chapel Hill, NC 27599 USA; 3 Upstream Thinking LLC, 1400 Lavaca Street, 8th Floor, Austin, TX 78701 USA; 4 National Institute of Medical Research, MITU, Isamilo Street, P.O. Box 11936, Mwanza, Tanzania

**Keywords:** Cash transfer, Economic empowerment, Sexual risk, Self-agency, Adolescent girls, Young women, AGYW

## Abstract

**Background:**

Transactional sexual relationships contribute to a high incidence of HIV infection among adolescent girls and young women (AGYW) living in low-resource settings. Cash transfers (CT) are a structural approach to reduce sexual risk behaviors, but their positive economic effects frequently fade after the program ends. We aimed to understand AGYW’s decision-making processes related to sexual, relationship, and financial decisions, in order to design a framework for a CT program that could lead to long-term financial independence and reduced transactional sex among AGYW.

**Methods:**

We conducted qualitative research with AGYW participating in a CT program in Tanzania. Phase one was formative research to understand the context and experiences of AGYW regarding sexual behavior, relationships, and finances. Participants included 36 AGYW (15–23 years old), 15 influencers of AGYW (mothers and male partners) and 10 financially empowered women (FEW – women aged 20–30 with a sustained, reliable source of income independent of their partner). Decisions and decision-making contexts of AGYW that we identified in phase one informed the content of phase two. In phase two we simulated scenarios for decision-making and economic goals with 80 AGYW and 40 FEW, in order to identify key principles or intervention opportunities to guide development of a CT program framework.

**Results:**

Through phases one and two of our research we identified three key themes in AGYW’s vision of their desired future economic state: 1) positive social image, 2) power balance and respect, and 3) emotional and economic security. An important theme distinguishing AGYW from FEW was that AGYW lacked a vision to build self-agency.

**Conclusions:**

Our findings suggest that providing economic resources to AGYW through CT without ensuring self-agency is unlikely to be an effective long-term intervention for economic empowerment. Using these findings we developed a framework for CT programs with three key pillars for developing self-agency: 1) emotional efficacy, to increase AGYW’s perception of rewards associated with developing self-agency; 2) social efficacy, to build constructive relationships and exit negative relationships that inhibit self-agency, and 3) economic efficacy, to help AGYW build a resilient stream of financial resources.

**Supplementary Information:**

The online version contains supplementary material available at 10.1186/s12889-020-10130-8.

## Background

Adolescent girls and young women (AGYW) are among the populations most vulnerable to HIV in Eastern and Southern Africa [1]. In Tanzania, over the past decade HIV prevalence has been twice as high among AGYW aged 15–24 years as among boys and young men of the same age [2]. Transactional sex (sex in exchange for financial or material goods) is considered an important driver of HIV risk among AGYW living in economically deprived settings [3]. One structural intervention that has been used to complement biomedical and behavioral HIV prevention programs is cash transfers (CT). CT hold the potential to reduce HIV risk behaviors [4–7], delay sexual debut [7, 8], and increase individual and household income and access to services, including schooling [7]. However, CT interventions which give money directly to AGYW have taken place within the context of time-limited randomized controlled trials [5, 6]. Once cash transfers end, AGYW are susceptible to reverting to previous HIV risk behavior if they lack skills and resources to maintain financial independence [9]. In order to sustain the positive effects of CT it is important to design interventions which improve AGYW’s financial capabilities over the long term.

The Sauti Project, funded by the US President’s Emergency Plan for AIDS Relief (PEPFAR) and implemented by Jhpiego, is a national initiative to provide health and HIV services to vulnerable groups in Tanzania, including AGYW. One of its structural interventions for AGYW, via the USAID-funded DREAMS program, provides CT as part of a larger intervention to reduce HIV incidence among AGYW through economic security and personal empowerment [10]. Sauti’s standard intervention package for AGYW includes: 1) biomedical interventions, including community-based HIV testing and counseling; 2) behavioral interventions, including peer-led education sessions to promote health-seeking behaviors by improving negotiation, self-efficacy, and condom-use skills; 3) a structural intervention called WORTH+, which includes entrepreneurial training, mentorship, and savings and loan groups to equip women with the necessary skills to plan their economic development more efficiently; and 4) cash transfers. Each eligible AGYW received a mobile phone, SIM card, and 70,000 Tanzanian shillings (TZS, approximately USD $31) via mobile money every 3 months over a period of 18 months. Eligibility criteria included being an AGYW between the ages of 15 and 23 years, being out of school, living in a participating village, completing 10 h of behavior change communication, and being willing and able to give voluntary, informed consent/assent to all study procedures, including HIV and HSV-2 testing and to receive the test result. Participants aged under 18 required the consent of a parent or guardian unless they met criteria for emancipated minors.

**Fig. 1:**
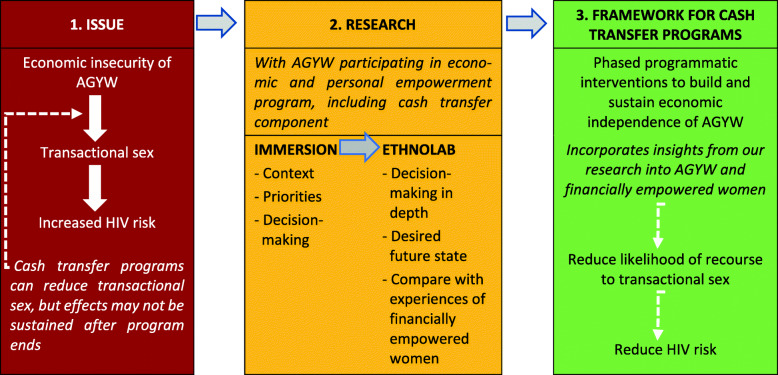
Approach to research with AGYW and developing a framework for cash transfer programs. Graphical representation of framing of research issue, research approach, and framework developed from research results

The purpose of this study was to 1) investigate the kinds of decisions that drove transactional sexual behaviors among AGYW in the Sauti Project; 2) understand how AGYW made those decisions; and 3) understand how CT could impact sexual, relationship, and financial decision-making in this context. Based on these findings, we developed a framework that can be used to design CT programs leading to long-term financial independence among AGYW, thus supporting HIV prevention goals by reducing the likelihood that AGYW will resort to transactional sex (Fig. 1).

## Methods

We conducted two phases of research. The first, Immersion, was formative qualitative research using in-depth interviews to develop a broad understanding of the context and experiences of AGYW participating in the CT intervention. Content analysis of the results from Immersion informed development of the second phase, EthnoLab. EthnoLab is a scenario-based behavioral decision-making game (in this case presented as a board game), with qualitative follow-up discussions, to understand the contexts, emotions, and mental models that influence decision-making. EthnoLab was developed by Final Mile Consulting and has been successfully applied in several projects across the African continent, India, and the US [11, 12]. Content analysis of the results of EthnoLab was then used to inform the design of a CT program framework (Fig. 1).

### Immersion

For this formative qualitative assessment, we interviewed a convenience sample of three categories of AGYW aged 15–23 years, engaged through face-to-face contact: AGYW who had received their initial CT within the past 2 months, plus the WORTH+ program (*n* = 18); AGYW who received WORTH+ but no CT (*n* = 12); and a control group of AGYW receiving neither intervention (*n* = 6). We also interviewed two groups that were not AGYW: influencers of AGYW (mothers and male partners) (*n* = 15); and financially empowered women (FEW), defined as self-employed or employed women aged 20–30 years with a sustained, reliable source of income independent of their partner (*n* = 10). FEW were included to understand the experiences of women who had traversed the adolescent journey in the same community in which our population of AGYW lived.

We were specifically interested in capturing decision-making processes of AGYW and FEW relating to sexual, relationship, and financial decisions. The qualitative interviews explored three areas: 1) What drivers do AGYW say lead to transactional sexual behaviors? 2) How do CT impact their decision-making in this context? and 3) What limitations may current CT programs have in achieving long-term impact (i.e. economic empowerment and supporting a reduction in transactional sex)? (For the interview questions, see Supplementary Material 1.)

Recruitment for the interviews was facilitated by community-based organizations implementing the Sauti Project. The Immersion phase lasted from June 2017 to August 2017. Interviews were conducted by trained female qualitative-research assistants from the National Institute for Medical Research, at locations including schools and local-government offices where a room could be arranged for the interviews to take place without interruption and without the presence of non-participants. Participants gave their informed consent in writing, and the purpose of the study was explained as part of the consent form. Interviews lasted about 60 min. They were conducted in Kiswahili, using an interview guide, and were audio-recorded, then transcribed and translated into English. Participants were given TZS 5000 for transportation costs for the interview. All participants completed the interviews, with no drop-outs.

To analyze the transcript data, we created a codebook reflecting question domains, and including concepts of emotional appraisal for decision-making (relevance, implications, coping potential, and social norm significance) derived from the Emotional Appraisal Framework [13] (Table 1).

**Table 1:** Codes for Immersion interview transcripts

1	Personal/life context	18	Perceptions about contraceptives
2	Goals/priorities/aspirations/motivation/role models	19	Perceptions about peers’ behaviors
3	Barriers to empowerment/challenges	20	Inflection points
4	Empowerment enablers/support system	21	Trade-offs/adjustments/compromises
5	Mental models/decision heuristics	22	Self-image/internal standards
6	Expectations/perceptions regarding partners/relationships	23	Reputation/social status
7	Dynamics of relationships/partners	24	Perception of risk
8	Family dynamics/relations/expectations	25	Perception of causality
9	Relations/interactions with community/local leaders/peer groups/religion	26	Advice and Influences
10	Awareness & knowledge levels/gaps	27	Emotions
11	Self-efficacy/skills	28	Perceptions of control and power
12	Access to resources – money, networks, information	29	Sexual behaviors & perceptions
13	Social norms/traditional beliefs & practices	30	Financial behaviors & attitudes
14	Conflict/negotiation/power dynamics	31	Information sources & cues
15	Mental accounting	32	Habit-building/routines/disruptions
16	Coping potential/strategies	33	Experience/impact of CT
17	Perceptions about cash transfers		

Transcripts were coded manually by seven of us in an iterative process: new themes that emerged during analysis of the transcripts were added to the codebook and the transcripts were then re-analyzed. Key observations and insights were grouped and summarized for each code. We then prioritized those observations and insights relating most clearly to sexual, relationship, and financial decisions and decision-making processes, as well as the impact of the CT program upon AGYW. These inflection points were used as an input to develop the EthnoLab (Table 2).

**Table 2:** Inflection points in AGYW decision-making

Sexual Decisions	Relationship Decisions	Financial Decisions
Pressured into sexual relationship by boyfriend	Being propositioned for a relationship by a peer male	Not having enough money for food
Being asked for sex by a boyfriend/ peer male	Being propositioned for a relationship by an older man	Facing an immediate need for money because of an unexpected event
Refusing sex	Not getting what she wants/expects/needs from boyfriend	Starting period and needing sanitary pads
Deciding to stop condom use	Learning that she is pregnant	Seeing items like clothes and shoes and wanting to have them
Asking boyfriend to take an HIV test	Discovering that boyfriend is cheating on her	Setting goal for oneself
Negotiating condom use	Taking on an additional boyfriend	Business revenue shortfall
Discussing family planning	Switching boyfriends	To start a business or not
Thinking about getting tested for sexually transmitted infections	Emotional stress in relationship influencing choice to leave/stay	Needing capital for business
Having sex with another woman	Physical violence in relationship influencing choice to leave/stay	To join CT program or not
Considering sex work as a means to gain money	Feeling like boyfriend doesn’t meet her financial needs/expectations	To share/loan money (to peers, family, or partner) or not
	Boyfriend leaves her for another girl	To stay committed to financial empowerment workshops or not
	Getting involved with bad role models/changing peer network	Creating a savings goal/target
		Deciding to save money
		Deciding that she will rely on herself rather than on a man
		Deciding that she will seek financial resources through methods other than sexual relationships

### EthnoLab

The objectives of the EthnoLab phase of research were: 1) to understand AGYW’s decision-making processes in greater depth, 2) to understand their desired long-term economic state, 3) to compare AGYW’s decision-making and aspirations with those of FEW, and 4) to identify key principles and intervention opportunities to guide development of a CT framework that could result in lasting behavior change for AGYW’s sexual, relationship, and financial decisions.

### EthnoLab scenario development

**Fig. 2:**
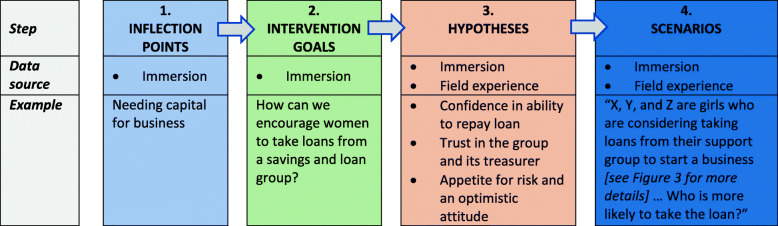
Four-step process for development of EthnoLab scenarios. Graphical depiction of stages in development of EthnoLab scenarios, showing data sources from the research stages, and giving examples of scenario questions

EthnoLab tests scenarios related to AGYW’s and FEW’s decision-making and its consequences for their financial independence, and thus potentially for their sexual risk-taking. These scenarios were developed in a four-step process (Fig. 2). Step 1 was to identify key inflection points – commonly occurring situations that can lead to a decision with either a positive or negative outcome for financial independence and/or sexual risk-taking. These inflection points were determined from the analysis of the Immersion interview transcripts.

In step 2 we identified intervention goals to be tested by EthnoLab, to see if they might lead to positive outcomes for AGYW faced with decisions at inflection points. Like the inflection points, the goals were derived from the transcripts of the Immersion interviews. Each goal was framed as a question. For example, if the inflection point is “Needing capital for business”, a question underlying an intervention goal would be, “How can we encourage women to take loans from a savings and loan group?” Step 3 was to develop several hypotheses, or potential answers, for each question, based primarily on data from the Immersion interviews, together with the researchers’ own field experience. In our example, these hypotheses were: confidence about being able to pay the loan back; trust in the group and its treasurer; and an appetite for risk and an optimistic attitude.

**Fig. 3:**
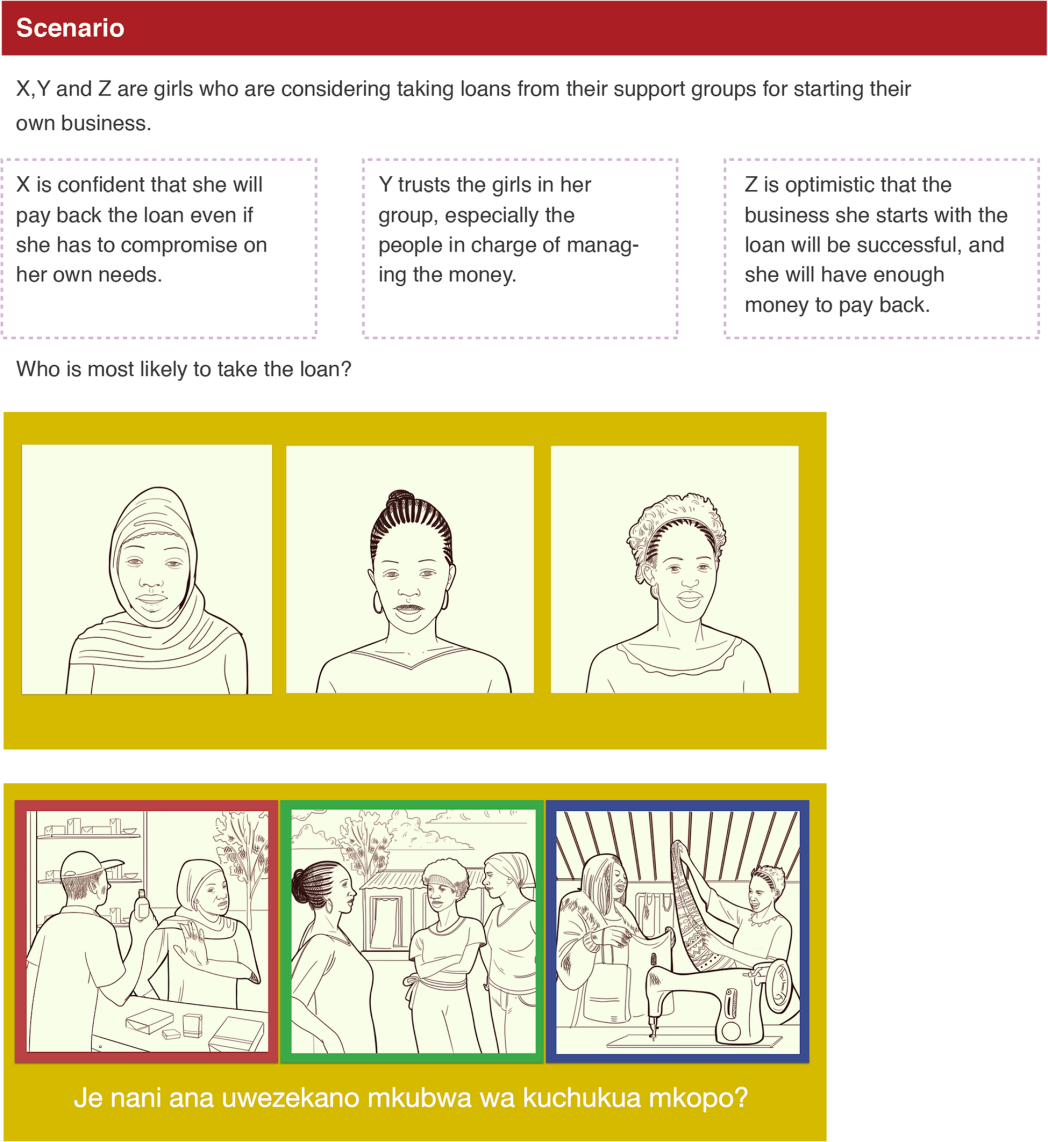
Example of an EthnoLab scenario. Scenario from EthnoLab, with drawings of three people and three scenarios as prompts for participants’ choices

In step 4, we developed scenarios – realistic narratives that encapsulated the inflection point as an AGYW or FEW might experience it, and that presented the three hypotheses as options for each EthnoLab participant to choose among (see Supplementary File 1). The fully developed scenario for our example is shown in Fig. 3.

For each scenario, an EthnoLab moderator used follow-up questions to facilitate a discussion with participants, in order to get a qualitative understanding of the decision-making process. Questions for the scenario shown in Fig. 3 included: 1) Have you ever borrowed money from someone? From whom? When did you pay it back? 2) What are the reasons for borrowing in your community? What discourages you from taking a loan? and 3) Do you plan to borrow from the support group? For what reason? These questions acted as prompts to elicit further information about inflection points – including ones that were not a participant’s first choice but that might also have some bearing on her decision-making.

Once the scenarios were designed, we reviewed them with our collaborators. Twenty scenarios related to sexual, relationship, and financial decisions were ultimately selected to be used in the EthnoLab (see Supplementary File 1).

### EthnoLab: decision-making board game

A total of 24 EthnoLab sessions were conducted in January 2018, with five participants in each session. Three distinct samples were recruited: AGYW who received CT plus behavioral interventions and WORTH+ (*n* = 60; 12 sessions), AGYW who received the behavioral interventions and WORTH+ but no CT (*n* = 20; 4 sessions), and FEW who had not received any interventions (*n* = 40; 8 sessions). Participants again gave their informed consent in writing, and the purpose of the study was explained as part of the consent form. All participants completed the interviews, with no drop-outs.

During EthnoLab, the five participants play a board game called “Flowers and Thorns”, lasting about 90 min. The familiarity of the board-game format and the incentive of winning increase participants’ engagement by creating an enjoyable environment that helps them relax, rather than feeling that they are being scrutinized.

In the game, players take turns to roll dice and move their game pieces around the board. Landing on a tile with a flower means completing an activity such as a simple puzzle or word game, which acts as an ice-breaker to bolster their engagement. When a player lands on a thorn, all players take part in one of the audio-visual scenarios. Each scenario simulates the participants’ real-world context by presenting a protagonist of the same age, gender, and socio-economic level in a situation that requires a decision or action. After viewing the scenario, all players are given three possible responses and asked to select one by secret ballot. The players are told that the response selected by the majority of participants will be considered the winning option. This format aims to reduce social desirability bias in the participants’ responses, by encouraging them to focus not on their own personal choice but rather on how they believe their peers think and act. The vote is followed by a 5-min group discussion that delves further into the theme and setting of the scenario (see Supplementary File 1). The game is played until all scenarios have been voted on and discussed.

The discussions in each EthnoLab session were audio-recorded and transcribed. We coded them using a process similar to the coding of the Immersion interviews, with codes derived from the Emotional Appraisal Framework; examples of codes include Preferences, Beliefs, Barriers to empowerment, Goal relevance, and Social norms.

The individually coded transcripts were merged and then disaggregated between AGYW and FEW. Matrices were used to synthesize and compare the content on sexual, relationship, and financial decision-making between the groups.

We examined the long-term economic state that AGYW desire and the decisions they are likely to make, and how FEW have achieved their own desired state. We compared the decision-making processes associated with sexual behaviors, relationships, and finances between the two groups. We then brainstormed potential program design strategies that could overcome barriers and enhance enablers of success.

## Results

### Immersion

The Immersion research helped us identify decisions and decision-making contexts experienced by AGYW that may impact their economic situation and their propensity to engage in transactional sexual relationships.

Two adolescent girls (one of whom was participating in the CT program and one of whom was not) described similar experiences of powerlessness to refuse their respective boyfriends’ demands for sex:

*“He was staying with his parents but he has his room outside (the main house). He told me (he wanted to have sex). I refused but at the end of the day I found myself accepting.”*

*“I told him that it’s too early, he refused (to accept my answer). I insisted (but) I had no other option because it was in his place, so I had to accept.”*

The girls reported that their boyfriends did not use a condom.

CT had some positive impacts on AGYW: they helped AGYW meet basic needs, somewhat reduced their financial dependency on men, and could help them resist the influence of peers who were more likely to spend their money for immediate gratification, or to engage in transactional sexual relationships. Statements such as the following were representative of AGYW’s responses about the positive effect of CT:

*“One of my friends, she used to depend on money from boys. ( … ) But when she received this 70,000 (shillings), she has told me that she doesn’t ask for money from boys (any more).”* Adolescent girl (15–19 years)

The CT and WORTH+ program had the effect of engaging young women in business who might otherwise be idle. One young woman explained the impact on herself compared with her peers:

*“(My friend) is free most of time – if she wishes to go to see someone she can go anytime. But I am not free so I don’t care about those things.”* Adolescent girl (15–19 years)

Another describes how her focus on developing her business changed her thinking about relying on a boyfriend for material support:

*“I have just one friend. But we have not seen each other in a long time. She used to tell me the things that (her boyfriend) would tell her and the things he would buy for her, but I did not desire it.”* Adolescent girl (15–19 years)

While the positive impact was encouraging, we inferred that it may not be sustained in the long term. For example, some AGYW said they saw CT recipients using the money to satisfy immediate material desires instead of investing it in something that could bring longer-term benefits:

*“I have not even seen them doing business, they are just buying clothes.”* Adolescent girl (15–19 years)

In addition, we noticed that even when motivated to succeed in business, AGYW may not be able to cope with unanticipated adverse situations:

*“(My) mother this year, month of April, she had been hospitalized. ( …) I was the one staying with her in hospital so all expenses were on us, so the business went down a bit.”* Young woman (20–23 years)

AGYW did not envision a path toward self-reliance and were likely to go back to men when financial needs arose:

*“Temptations do occur. It’s like if for example you don’t have money. So you start to request for money and later he will tell you to pay him back. It’s like that – you will pay him by having sexual intercourse with him.”* Adolescent girl (15–19 years)

We concluded that men may continue to be a channel for money for AGYW, and AGYW may revert to engaging in transactional sexual relationships upon ending the CT program. This means that overall, time-limited CT programs may not be sufficient for AGYW to build lasting economic stability independent of male partners. Immersion results, including the example presented here, served to inform the development of the EthnoLab scenarios.

### EthnoLab

The EthnoLab research used the findings from the Immersion phase to explore the long-term economic state that AGYW desire, and the opportunities for interventions to foster long-term economic independence, and thus influence behavior change relating to transactional sex and HIV risk. The EthnoLab also sought to learn from FEW, who have already achieved long-term financial independence from men.

### AGYW’s vision of their long-term economic state

From the EthnoLab analysis, three themes emerged in AGYW’s vision of their desired economic state: 1) positive social image, 2) power balance and respect, and 3) emotional and economic security.

#### Positive social image

This refers to achieving and maintaining a positive social image. AGYW feel a heightened need to be admired in the community they live in, and especially by their peers. Participants frequently highlighted social interactions, such as the one below, when an AGYW was asked about her entrepreneurial experiences:

*“(If) they have seen that one (girl) has not sold (anything) since morning until evening, there are others who will start to laugh, and (…) they will not respect her.”* Adolescent girl (15–19 years)

Participants reported that social comparison and wanting to look good to their peers directed their decision-making about how to use their CT. In some cases, this led them to use the cash in ways that were not necessarily in their financial long-term interest:

*“Because she has seen others looking good...and if she stays with those girls who have partners for money, she will be tempted by those girls to do the same” *Young Woman (20–24 years*)*

Perceptions of societal norms around who should start a business negatively impacted some AGYW’s confidence about using CT constructively:

*“It is just a belief we have that matured [older] people are supposed to do business and gain profit, but for us young girls, we cannot gain anything. So that belief has affected us, because if you want to start a business, you first look at your age; you cannot stop yourself thinking that you’re still young, because business is for matured people.”* Young woman (20–24 years)

However, for others, social comparison had a positive impact on AGYW’s decision-making, by inspiring them to start a business of their own:

*“I like to do a business of selling rice at the rice mill. I like to look at my friend; my friend is a good businessperson.”* Young woman (20–24 years)

In summary, a positive social image is an essential future state that AGYW want to achieve. It tends to influence their behaviors, including their use of the money received as part of the CT program.

#### Power balance, and respect in relationships and the community

AGYW expressed the desire to gain respect, both within their relationships and in the community. This was expressed in terms of the ability to make some decisions without needing to seek permission from men, and to avoid humiliation by a partner:

*“For me, I think it is good because I earn my own money. I can buy anything I need, because when you depend on men, sometimes they disrespect you, especially for us without high education.”* Young woman (20–24 years)

Facing challenges and remaining firm during setbacks was another way AGYW attained respect in the community:

*“As for me, I see every job has its challenges, (…) so if you lower yourself, you will not be respected, but if you remain firm, no one will joke with you.”*

Based on their own experiences or observations, AGYW believed that a woman who can contribute toward the household income could be respected in the community. Increased respect from a partner might also lead to a more equitable relationship. While AGYW acknowledged that a wholly balanced relationship might not be achievable, gaining more respect and power in relationships was a desired future state. A girl in the control group (i.e., who was not participating in the CT program) imagined how having independent funds to start a business might affect the dynamics of her relationship:

*“Most of the time I will be in my business, but if you don’t have business you will be thinking, let me go to my boyfriend. He might ask, why are they are sending you money, for what and who are you, then he will use force to take money. When he will ask me, I will tell him that first they have decided to help and rescue me from my situation, for example if I do business, I can help you, even it can help me and my parents.”* Adolescent girl, (15–19 years)

#### Emotional and economic security

AGYW expressed their need for security in several ways. A sense of emotional security derived from a romantic partner and long-term economic stability was an important pursuit. AGYW sought partners who strengthened their feelings of self-worth and emotional security. Sometimes this was found in an older partner:

*“To be with a person who is older than you and he recognizes your value, teaches you how to live, and makes you learn many things from (him).”* Young woman (20–24 years)

Long-term relationships were another route to gain emotional security, with some AGYW preferring partners who they believed would commit for the long term:

*“I will accept because that person has been through everything, so when they are with me, they will settle down.”* Adolescent girl (15–19 years)

AGYW also frequently expressed the need for economic security, with access to material comforts that surpassed basic needs. In the absence of alternative means, male partners tended to be the default route, and AGYW sought partners who demonstrate the potential to satisfy their material needs and wants:

*“Some (AGYW) need money, others need someone to pay for their house rent, one for outings, and another one for buying food.”* Young woman (20–24 years)

Our analysis of the EthnoLab research indicated that CT programs may be able to meet some immediate economic needs and disrupt the common route of seeking relationships with men, at least in the short term.

### Self-agency among financially empowered women (FEW)

An important theme that emerged from our research with women who have managed to sustain a reliable income source independent of their partner is a vision to build self-agency, i.e. the ability to make strategic choices and control resources to meet their interests. FEW exhibited this in various ways. They expressed the need to be independent and generating income on their own. They also described maintaining a degree of self-control to avoid being dependent on men. FEW recognized that attaining economic self-agency may be a prerequisite to achieving respect and power in their sexual relationship with their partners:

*“(It is important to be able) to ask a man to use a condom, but also, girls, we must not have a desire for money, we must have a small business, because if you have your many businesses, you can (choose) not to accept any man with any amount, ten thousand, twenty thousand. You can stand against him and show him you have a place you can get that money.”* Financially empowered woman (20–24 years)

Another woman also reported achieving greater equity in her relationship as a result of her business:

*“For now, he hasn’t (complained). I have started seeing the profit and he has trusted me and I am trustworthy. I give (at home) and he gives, just like that, we help each other, you know life.”* Financially empowered woman (20–24 years)

To attain self-agency, a critical step was setting attainable and achievable goals:

*“I started roasting groundnuts. Later, I had a spot where I sold coffee, ginger, milk. (…). It is just goals. If you have goals, like I started roasting egg groundnuts. I roast them and take them to a shop. (…) I said, ‘With this money I will be given, I will start another strategy.’ When I received the money the first time, I started buying fruits from the market and hawking [selling] juice.”* Financially empowered woman (25–29 years)

To achieve these goals, FEW developed business and interpersonal skills organically, by observing others and learning from their own as well as others’ mistakes. FEW were willing to involve their social network to help them plan their business activities, receive support during business setbacks, and get the courage to persevere:

*“You cannot run your business in harmony: someone is telling you not to do this and this. To get supported is something good. When you are supported you get strength and confidence.”* Financially empowered woman (25–29 years)

However, FEW who lacked this support structure relied on their internal beliefs, confidence, and ability to deal with failure:

*“God likes people who dare. Even if you (…) fail, you did it!”* Financially empowered woman (25–29 years)

## Discussion

We conducted qualitative research with AGYW who are vulnerable to transactional sexual relationships that place them at risk of HIV. The first phase of our research (Immersion) provided insights into the impact of the Sauti Project’s CT program upon AGYW’s economic independence, and into the factors that shape their decisions in three key areas of sexual behavior, relationships, and finances. In the second phase (EthnoLab), we explored how AGYW see their desired economic state for the long term, which they characterize as one of positive social image, power balance and respect within their relationships and in the community, and emotional and economic security.

Maintaining a positive social image, especially among one’s peers, is well documented among adolescents in general [14–16]. As a desired future state, it is not necessarily negative, but the pursuit of social status in the short term can lead to problematic behavior (e.g. engaging in multiple relationships, or spending one’s CT money on clothes) at the expense of long-term benefits [16]. However, the desire for a positive social image may also prompt AGYW to start a business in defiance of social expectations around gender, age, and roles. The example of others who are attempting or succeeding in business can act as an inspiration.

While AGYW do not expect to attain a completely equitable relationship with their partners, they do seek to improve their status and respect. They understand that women who contribute toward household income may be respected in the community, and that they can improve the power dynamic in their relationship by reducing their financial dependence on their partner. CT programs that also support income-generating activities are aligned with this long-term vision. Although research perspectives differ on whether men may react with hostility to their female partners’ economic empowerment and independence [7], the AGYW participating in the CT program, and the FEW, did not report such hostility during the EthnoLab discussions.

For many AGYW, the default route to emotional and economic security is relationships with financially secure men. CT programs can disrupt this dynamic, at least in the short term. But the default of dependence on men appears more viable than self-reliance, based on AGYW’s perceptions of themselves and their context. This is understandable, given that examples of FEW are relatively rare, and the path to self-reliance is a risky one, whose rewards seem less certain than those that can be gained from a relationship with a man. Many AGYW don’t consider their present life skills to be transferable to running a business, and their lack of experience gives them little certainty about the feasibility of self-reliance.

The primary theme that emerged from our interactions with FEW – women who have achieved and sustained a state of economic security and independence from men – is a vision to build self-agency. Empowerment literature describes self-agency as an ability to make strategic choices and control resources to meet one’s interests [17]. For FEW, the path to self-agency consisted of setting attainable goals to ensure a consistent income source; gaining business and interpersonal skills from observation of others and leaning from their own mistakes; and getting support from social networks for planning their businesses, coping with setbacks, and encouragement to persevere.

Comparing the findings from AGYW with those from FEW, it becomes clear that self-agency is missing from AGYW’s vision of their desired future state. While the Sauti project provided elements important to economic independence, including the WORTH+ structural intervention and CT (alongside interventions directly targeting sexual risk and HIV), our research suggests that providing resources, including CT, to AGYW without interventions to build their self-agency is unlikely to be effective long-term. This is congruent with other research into CT programs, which has found that any short-term behavioral improvements such as reductions in early marriage, total live births, and HIV infection fail to translate into improvements in the long term [9].

**Fig. 4:**
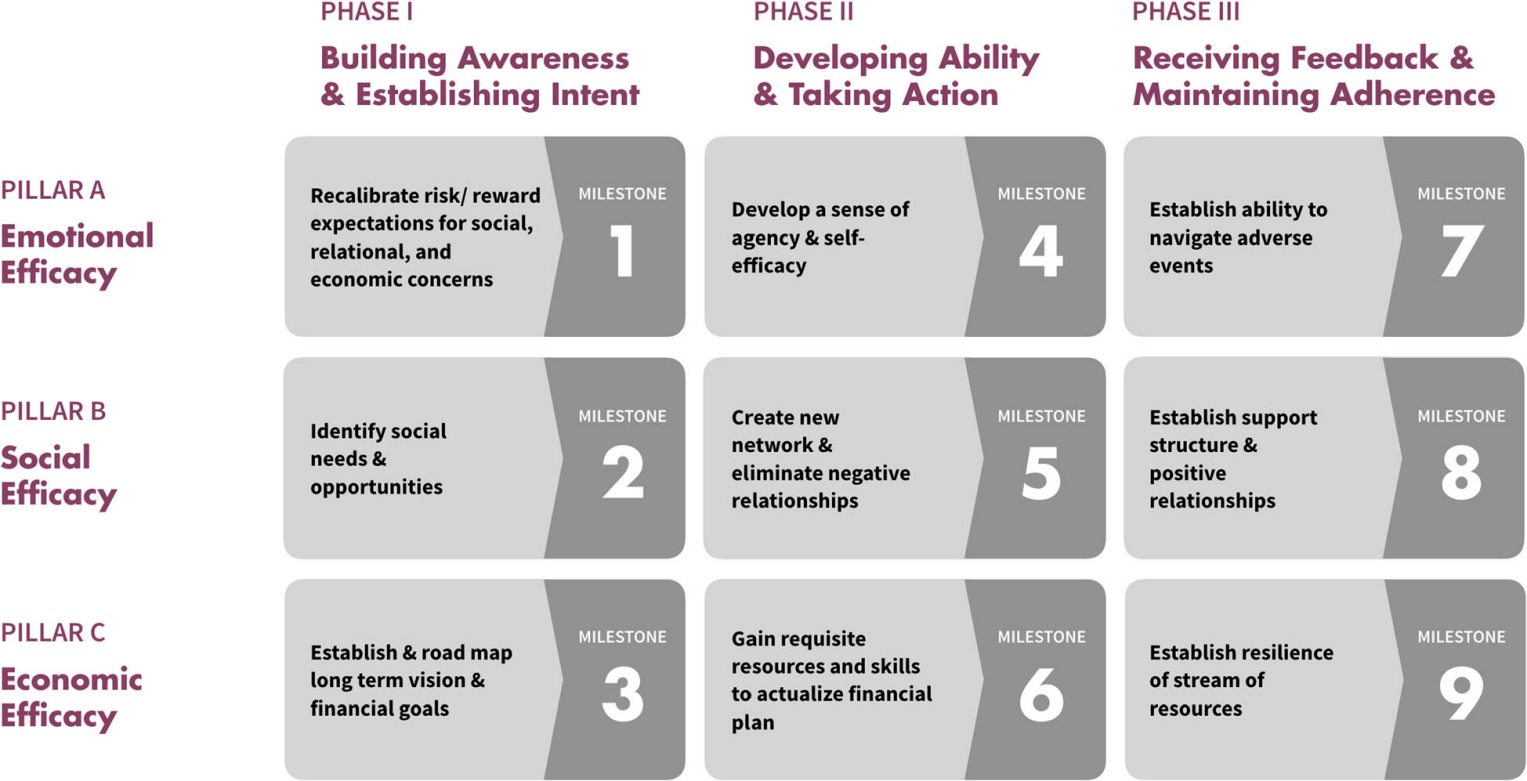
Framework for CT programs for AGYW. 3X3 framework showing pillars and phases of cash transfer program, with milestones for each pillar in each phase

We recommend that building self-agency among AGYW should be a key objective for a CT program to help AGYW achieve long-term financial independence and lower their vulnerability to behaviors that put them at risk of HIV. We therefore developed a framework which can serve as a high-level reference check to evaluate existing CT programs and inform the development of new ones (Fig. 4). The framework proposes three “pillars” of self-agency: emotional efficacy, social efficacy, and economic efficacy. These pillars support AGYW’s vision of their desired future state (positive social image, power balance and respect, and emotional and economic security), but they reframe these to incorporate components of self-agency. We envision that each pillar is developed over three phases: 1) Building awareness and establishing intent, 2) Developing ability and taking action, and 3) Receiving feedback and maintaining adherence. In each phase of the program, a programmatic milestone is proposed for each pillar as the basis for developing interventions. These pillars and phases are based on our understanding of adolescent decision-making, including maturity levels, risk and reward evaluations, and influence of social stimuli [16].

### Emotional efficacy

Emotional efficacy is core to self-agency. This pillar aims to help AGYW redefine the idea of positive social image and provide an alternative path to a positive social image. Research on behavior change among adolescents suggests that instead of relying on education-based interventions, changing the context in which risky behavior occurs may be more effective [16]. Information from social neuroscience also suggests that adolescent brains are more sensitive to rewards than risks [18]. The programmatic elements of this pillar therefore focus on changing the risk/reward trade-off among AGYW by increasing their perception of the rewards associated with long-term self-reliance, and developing a sense of self-agency by equipping them with the ability to deal with adverse situations.

### Social efficacy

Social efficacy is supplementary to emotional efficacy. It refers to the positive and negative social-contextual factors that facilitate or hinder the development and maintenance of emotional efficacy. Learnings from FEW suggest that social support can be an essential enabler in building self-agency and dealing with setbacks, and empowerment literature recognizes the ability of social connections to improve an individual’s resistance to transactional sexual relationships, avoid HIV risk, and increase resilience to recover from shocks [19]. The focus of the social efficacy pillar is therefore to increase the AGYW’s respect and power balance by building her capacity to identify and potentially exit relationships where she lacks respect and power, and identify and avoid negative peer influences. This pillar also helps her build constructive social relationships (with peers, mentors, and role models) that will motivate her to stay on the path toward long-term self-reliance. Constructive social networks also contribute to the emotional efficacy that AGYW need.

### Economic efficacy

Economic efficacy is also supplementary to emotional efficacy. It accounts for the functional skills required to graduate from dependence to self-reliance. The essential steps identified by FEW include setting achievable goals and acquiring the necessary business and interpersonal skills. Interventions such as providing life skills and vocational training have been tested successfully in other adolescent-focused programs, such as increasing engagement in income-generation activities and reducing teenage pregnancy and early marriage rates [20]. This pillar therefore focuses on helping AGYW establish a personalized financial plan and gain the skills to actualize it, in order to build a resilient stream of financial resources and fulfil her need for economic security and comfort. The CT element of financial empowerment fits well within this pillar since it can enable and accelerate these economic goals.

### Programmatic implications

This framework suggests concepts related to emotional, social, and economic efficacy that could be integrated within existing empowerment programs for AGYW to complement the CT component. CT programs that acknowledge the desires and goals of AGYW, while helping them develop the attitudes, expectations, and decision-making processes associated with FEW, stand a greater chance of helping AGYW build long-term financial independence. Our framework does not ignore the need for developing financial and business skills, and access to resources, that is central to many CT programs, but it focuses on a wider range of behavioral indicators, and the milestones are intended to help AGYW develop behaviors that will lead them to long-term self-sufficiency. This in turn can reduce their need for transactional sexual relationships, and thus lower their HIV risk. To serve as examples, some suggestions for elements of such programs are listed here, keyed to several of the milestones in the framework shown in Fig. 4.

#### Recalibrate risk/reward expectations (milestone 1)

Create platforms and events that disseminate stories of women who have successfully created alternative incomes, as well as those who have been negatively impacted by their dependence on men.

#### Create new networks and eliminate negative relationships (milestone 5)

Provide AGYW with easy shortcuts to categorize different relationships, identify gaps in their support network and find strategies that help them cope emotionally and economically when they exit negative or transactional sexual relationships.

#### Gain requisite resources and skills to actualize financial plan (milestone 6)

Engage FEW as mentors for AGYW to advise them on how they might achieve their personal goals. An apprenticeship program that allows young women to work alongside successful businesswomen could give them real-life business skills. AGYW could also be given a list of occupations to inspire new business ideas, rather than the limited ones (e.g. soap-making) that they may typically be exposed to. While there may be a limited number of FEW in a given community, they may be able to provide expanded support through their own business and social networks; and male business owners could equally teach skills and encourage program participants, even if they cannot serve as models of successful female participation in business.

#### Establish ability to navigate adverse events (milestone 7)

A mentorship program can go beyond helping AGYW build the practical skills they need for economic self-sufficiency. It can intentionally address the building of self-agency and prepare AGYW for the realities of business setbacks, helping them frame failures as a positive opportunity to learn and grow.

### Limitations

This research used a convenience sample of AGYW who were primarily part of the Sauti CT program in the Shinyanga region of Tanzania. In the Immersion phase, the sample of AGYW that had not received any interventions was not large enough to allow comparisons between the effect of receiving CT or no CT on each of the decision-making areas of sexual behavior, relationships, and finances. However, a strong effort was made to achieve a representative sample of AGYW with varying levels of sexual experience, exposure to different levels of interventions, and age groups. We believe that the insights and implications are relevant beyond the population and geographic focus of the study, in part because the study design leveraged insights into CT programs and AGYW decision-making from research in other contexts (4–8). We have not had the opportunity to test our framework by seeing it implemented within a CT program, but beyond offering a template for a complete program, it is intended to serve as a way of assessing potential gaps within existing or planned programs.

## Conclusion

The study used a unique qualitative research design and methods to understand the current and projected decision-making processes of AGYW. Our research identified that while financial resources such as CT can act as enablers of economic self-reliance in the short term, they are insufficient as a stand-alone intervention in the long term. Self-agency, an essential component for sustainable empowerment which we learned was a key theme among FEW, is currently missing in AGYW’s vision of their future state. This insight implies that a CT program that does not address self-agency may not have a long-term impact on AGYW’s economic self-reliance, and is thus unlikely to lead to behavior change around sexual risk-taking.

To achieve long-term effectiveness, we propose a framework for future CT transfer programs. Its three pillars of emotional, social, and economic efficacy support AGYW’s vision of their future state, but reframe it with phased programmatic elements that build self-agency. The phased approach moves the program beyond engaging the interest of AGYW (Phase I) and building their skills and encouraging action (Phase II). We envisage achieving a sustained impact through supportive relationships and networks (Phase III) that provide feedback and help AGYW to stick to the path that will lead them to economic self-reliance, making them more able to reduce their dependence on sexual relationships that can put them at risk of HIV.

## Supplementary Information

**Additional file 1.** Immersion and EthnoLab Research Questionnaires. Discussion guide for Immersion phase of research (pp.1–12), and scenarios and discussion questions for EthnoLab phase of research with AGYW and financially empowered women (pp.13–37).

## Data Availability

The datasets used and analysed during the current study are available from the corresponding author on reasonable request.

## Abbreviations

AGYW: Adolescent girls and young women
CT: Cash transfer
FEW: Financially empowered women
HIV: Human immunodeficiency virus
HSV-2: Herpes simplex virus-2
PEPFAR: US President’s Emergency Plan for AIDS Relief
TZS: Tanzanian shilling
USAID: United States Agency for International Development
USD: US dollar
