# Supplementary material for: Developing a framework for cash transfer programs that foster sustained economic empowerment to reduce sexual risk among adolescent girls and young women: a qualitative study
Source: BMC Public Health. 2021 Jan 11;21:122. doi: 10.1186/s12889-020-10130-8 (PMC7802135; doi:10.1186/s12889-020-10130-8)
Supplement: Supplementary file 1 — Additional file 1. Immersion and EthnoLab Research Questionnaires. Discussion guide for Immersion phase of research (pp.1–12), and scenarios and discussion questions for EthnoLab phase of research with AGYW and financially empowered women (pp.13–37). [file 12889_2020_10130_MOESM1_ESM.pdf]

IMMERSION RESEARCH QUESTIONNAIRE

Baseline BE interview guide

1. Adolescent Young and Married Women
Interview Format – Using a semi-structured discussion guide with contextual scenarios and memory prompts.

Thank you for taking the time to talk with me today. I would like to interview you for 1 **hour** and this will be more like a discussion.

You can draw from your own experience as we go through some of the stories and discussions. We are interested in understanding the goals, experiences and expectations of women living in the community. We would like to also learn about your social relationships, health decisions and financial decisions. You are welcome to express your genuine thoughts and experiences. There are no right or wrong answers.

Before we begin, do you have any questions for me?
(Rapport Building Conversation)

<u>Context Setting</u>:

1. Can we start with learning a little about you? Tell me a little about yourself:
   a. What part of town do you live in?
   b. Do you live with other people (family/ friends/ boyfriend)?
   c. Siblings?
   d. Where did you grow up?
2. What does your typical day look like?
   a. What do you do on a daily basis?
   b. What do you do during your free time? (What they do for fun)
3. Do you have a boyfriend?
   a. Are you pretty serious?
   b. Does he take care of you?
4. What are your plans/hopes for your future?
   a. What kinds of activities are you involved in that move you closer to those hopes?
   b. Are there particular people that help you move closer to those hopes?
      i. What do those people do to help?
   c. Are there people that might be preventing you from moving towards your hopes?
      i. Who/ Why/ How?
   d. Are there aspects of society, or life in general, that are preventing you from moving towards your hopes?
      i. What/ Why/ How?
   e. Do you have any people that you look up to/ any role models?
      i. If yes, who and why? What do you admire about them?
5. Do you feel that your ambitions are realistic? Why? if out of reach? Why?
6. What are some of your concerns in life, right now?

    a. How are you currently managing these concerns?
    b. Who do you normally discuss your concerns with? Why them?
7. In what ways are your experiences similar/different to those of other girls like yourself?
8. In what ways are your ambitions similar/different to those of other girls like yourself?
9. As part of recruitment for this interview, we asked about your personal sexual relationship behavior (Segments: 1. virgin/ late debut, 2. peer male relations only, 3. IG relations experience).

Can you tell me a little about the factors that have influenced your choices?

- a. Virgin/ Late debut:
    - i. What are some of the reasons that you've waited/ abstained from engaging in sexual relations?
    - ii. How has your experience been different than your friends?
    - iii. Has your experience been more difficult/ easier than your friends? Why/ How?
- b. Peer Relations Only:
    - i. What might be some reasons for only dating men your own age?
    - ii. Do you have friends who date older men?
        1. Have you considered dating older men?
        2. Are there trade-offs to dating older men? Why/ Why not?
- c. IG Relations:
    - i. What are some of the differences you've experienced between dating older men and men closer to your own age?
        1. (Explore the trade-offs as they are presented)
    - ii. Do you have friends who abstain from sex, or who choose to date men of their own age only?
        1. What do you think about their choices?
            - a. What kinds of pressures do they face?
            - b. How do they deal with those pressures?

<u>Cash Transfer Program Discussion</u>

(For YW who are part of the CT programs)
I understand that you've been participating in a program that has offered some classes that cover various aspects of life related to relationships and money management. I'd like to discuss some of your experiences so far with these classes, and what your reaction to those experiences has been. Your responses will not be shared with those who are conducting the programs, and your responses will not, in any way, influence your continued participation or benefits you may receive from those programs. We are asking for the benefit of future participants so that we might improve the programs to better suit the expectations and concerns of YW like yourself.

1. Let's start with some of the materials that you may remember from the workshops you've attended: (Here, materials from WORTH+ will be shared for review, to prime the following conversation).
    a. What aspects of the WORTH+ program do you remember most?
        i. What aspects stand out to you as most important to you? Why/ How?
        ii. Were there aspects of it that you felt were irrelevant or unrealistic?
        iii. How do you envision these kinds of activities helping you to achieve the plans and hopes you already had for yourself?
            1. Have they sparked new hopes/ plans?
    b. Let's talk about some of the specific topics (if they've already been through them in the program)
        i. Community Savings
        ii. Community Loans
        iii. The Road to Wealth/ Pursuing a business
    c. For each:
        i. What do these recommendations mean to you?
        ii. Are you comfortable with these ideas?
            1. Can you imagine participating in activities like these, or are they beyond what you believe to be possible?
                a. Why/ How?
            2. What kinds of support do you think you'd need to make these activities possible?
            3. What kinds of barriers do you think you might face in trying to participate in activities like these?

2. Let's shift gears and talk about some of the relationship based materials: (Here, materials from SBCC will be shared for review, to prime the following conversation).
    a. What aspects of the SBCC program do you remember most?
        i. What aspects stand out to you as most important to you? Why/ How?
        ii. Were there aspects of it that you felt were irrelevant or unrealistic?
    b. Let's talk about some of the specific topics (if they've already been through them in the program)
        i. Gender & Power Issues
        ii. Understanding Sexuality
        iii. Risky Behavior
        iv. Contraception
        v. Respectful Relationships
    c. For each:
        i. What do these stories and recommendations mean to you?
        ii. Are you comfortable with these ideas? Why/ How?
        iii. How relevant do you feel that these stories are to you and your personal life/ your decisions?
            1. Do you feel like you have any confidence that you might be able to follow through on any of the recommendations? How/ Why?
            2. What are some of the implications of doing so?

3. Finally, let's discuss the cash component of the program (DO NOT DISCUSS THIS SECTION WITH THE CT CONTROL ARM SEGMENTS)
    a. Can you tell me a little about the Cash Transfer component of the program?
        i. How does it work?
        ii. How is it working for you?
            1. How do you receive the cash?
            2. What are some of the implications for how this works for you?
        iii. How have others in your life responded to this program (family, friends, boyfriends, etc)
            1. What do you think about these reactions?
        iv. How do you think you intend to use the money?
            1. Are you comfortable with this arrangement?
            2. Are you confident in managing the money?
            3. Do you have any concerns regarding the money or the program?
                a. Do you have any concerns with how the money might make a difference on the relationships in your life?
        v. What about other girls, like you? Do you think they're experiences have been similar?
            1. What kinds of pressures are they facing with this program?
            2. What kinds of ambitions do they have to make the most of this program?
    b. What's your impression of the people who are implementing the program?
        i. Are there any issues of trust?
        ii. What do you understand of their motivations to be running programs like these?
        iii. What kinds of changes do you think they're going to have on your community, at large?
            1. Will these programs (have these programs) changed the dynamic of interaction between people in your community (between friends, family, boyfriends, etc)?
    c. What about the method of delivering the money? I understand that the money is delivered through a mobile phone.
        i. Have you ever managed money this way before?
        ii. How does that work for you (good/ bad)?
        iii. Are you anticipating/ experiencing any challenges with regard to how the money is being delivered/ managed?

QUESTIONS FOR TRUE CONTROL SUBJECTS

1. Are you familiar with social programs for young people, in particular, programs that deal with reproductive health, family planning, sexual behavior, and/ or money management?
    a. Can you tell me a little bit about some that you might be familiar with?
        i. Do you have any direct, personal experience with any? (probe)
        ii. Do you know of close friends who have had direct experience? (probe)

iii. Or, is your experience only from mass media? (probe)

I’d like to discuss a hypothetical program and get your reaction to it:

2. Let’s say there’s a program that provides workshops on relationship dynamics between young women and the various sexual relationships she might have, as well as money management and work opportunities she might pursue. As part of this program, the young women who participate would also receive an income of 70,000 TZS once every 3 months.

    a. What might be the ways she would use this money? How long do you think this money will last?
    b. Will this have any difference on her goal of feeling financially secured? Is it big enough? Why or why not?
    c. What are the typical expenses of women of her age?
    d. Do you think she would spend all the money that she received?
    e. Would she save any money for later? Why or why not?
    f. Do you think this will make any difference/affect her relationships especially sexual relationships?
    g. Would this make any difference on the need to have sex or engage in sex with older men?

3. Probe around Financial management:
    a. Have you or someone you know received this kind of income recently?
    b. How did you become aware of this?
    c. What were your initial thoughts before participating? Did you have any concerns?
    d. What’s your impression of the organizations who are implementing these programs?
    e. Do you think participation will make a difference in your life in any way? How?
    f. What have you done with this money?

Parents

Interview Format – Using a semi-structured discussion guide

Thank you for taking the time to talk with me today. I would like to interview you for 1 **hour** and this will be more like a discussion.

You can draw from your own experience as we go through the discussions. We are interested in understanding the goals, experiences and expectations of people living in the community. We would like to also learn about social relationships, health decisions and financial decisions in this context. You are welcome to express your genuine thoughts and opinions. There are no right or wrong answers.

Before we begin, do you have any questions for me?

Context Setting:

1. Can we start by telling me a little about yourself and your family?
    a. Where did you grow up?
    b. Siblings, friends, etc
    c. Kids
    d. Work/economic activity
2. What are your major life ambitions & concerns?
3. What kind of hopes do you have for your children?
    a. What might be your major concerns?
    b. Are they the same across all your children? Why/ Why not?
4. What kinds of hopes do you think your children have for themselves?
    a. Are these realistic?

Perspective on Cash Transfer/ Social Programs

5. There are various social campaigns underway to help mitigate risky sexual behavior among. Are you aware of any of these? Which ones?
    a. What's your impression of them?
    b. Do you know anyone who has participated in them directly?
    c. Have you seen any difference in behaviors because of these programs?
6. What impact do you see with these programs having? Probe re: familiarity with CT programs, specifically, if not already discussed.
    a. What's your impression of a program designed to help young women gain financial independence?
        i. For young women to be given cash as a way to reduce their dependence on men, for resources, and to reduce the likelihood of risky sexual behavior?
        ii. For young women to have more economic opportunities, to become less reliant on men for resources, in general, and to be more self-sufficient?
            1. Are these realistic goals for young women? Why/ How?
    b. What impact have you seen/ do you imagine a Cash Transfer program having on the power dynamics between young women and the rest of the people in their communities?
        i. Between peer males
        ii. Between peer females
        iii. Between IG males
        iv. Within families
        v. In the market/ employment

Cash Transfer Program Implementers
Interview Format – Using a semi-structured discussion guide

Thank you for taking the time to talk with me today. I would like to interview you for 1 **hour** and this will be more like a discussion.

Please note that we are not evaluating you or the program with this interview. We are hoping to learn from you, in your own words, and in your own judgment, about various aspects of the program and its influence on the young women, their families, friends and others in the community. This discussion, and any responses you provide will remain anonymous.
Before we begin, do you have any questions for me?

a) General Relationships with Students:

1. Tell me about your role as a CT implementer
    - Previous experience working with youth/ young women (YW)
    - Time with CT program?
    - Markets served?
    - What are your primary/ secondary responsibilities?
    - Number of workshops given?
    - How many YW have you interacted with/ seen go through the program?
    - How would you characterize the relationship you have with the girls who go through your program?
        - How, do you imagine, the girls would characterize their relationship with you?

b) Perceived relational and sexual norms:

1. One of the primary purposes of the program is to reduce YWs' dependence on men, in particular older men. I'm wondering if you can tell me a little about how YWs' relationships with older men (whether strictly financially based, or romantically based) are viewed by:
    - The YW themselves
    - By their male peers
    - By the YWs' families/ parents
    - Community

c) Perceived program relevance (as expressed by AGYW, families):

1. What do you see as the motivating drivers for AGYW who are participating in these programs?
2. What do you see as the barriers for AGYW who are choosing not to participate?
3. How is the program received by families (perceptions, attitudes, expectations)?
    - I understand that this may be the first monies that these YW will have of their own. How has this influenced the YW's parents, siblings, etc?
    - How has this affected the YW's peer relationships?
4. Do you see differences among YW by:
    - Age
    - Education (still in school vs. dropped out)

d) <u>Perceived program concerns (as expressed by AGYW, families):</u>
   1. What are the most common questions/ concerns you hear from YW (and family members)?
   2. Are there common patterns of questions/ concerns that YW raise during workshops?
   3. Are there common patterns of questions/ concerns that YW raise throughout the duration of the program?

e) <u>Perceived program efficacy:</u>
   1. What aspects of the program appear to be working most strongly?
   2. What might you improve about the existing program?
   3. Are there things that you would recommend doing that are not part of the existing program?

f) <u>Perceived program hurdles to effectiveness (as expressed by AGYW, families, implementers):</u>
   1. What kinds of things do you hear about the program that may be infeasible, or unrealistic to expect of YW?
   2. Are there aspects of the program that should be in place, but are not?
   3. Do you see any resistance to the program from family members, peer groups, boyfriends, etc?
   4. Have you seen any increase in risky behavior that can be attributed to the CT program? (now that the YW are suddenly flush with cash…)

g) <u>Usability of the program materials/ cash:</u>
   1. The money is currently delivered through cell phones, once every quarter the YW receive Tsh 70,000. How does this structure and timing work with the YW?
      - Are they able to access this format without problem?
      - Might the timing be better/ different?
      - What about the amount that they're receiving?

h) <u>Perspectives on the entrepreneurship aspects of the program:</u>
   1. How well do you think these align with the girls' expectations of themselves?
   2. How do the families/ friends/ peers view this component?
      - Is it reasonable to expect that these YW will use this money in a business building capacity?
      - Is this the first time these YW will be managing money?
      - How confident do you see these YW in following through on starting businesses with this money?
      - How motivated do the see these YW in using the money for business purposes?

i) <u>Concerning the attitudes of others and the influence of these programs on their behavior:</u>
   1. How do you see the CT programs influencing IG men and their behavior?

- Do you think that they're aware that the programs are intended to reduce YWs' dependence on them?
- How have they reacted (or how do you imagine they will react)?
- Will they find a way to take advantage?

---

2. Adolescent Young and Married Women – Medium Testing
Interview Format – Testing of different game formats and using a a semi-structured discussion guide to gather feedback

Thank you for taking the time to talk with me today. This session is expected to be 1 **hour** and we would like to keep it interactive.

Today, we are testing a new game and are interested in your feedback about it. The idea is to understand if you find the game fun and easy to understand. You are welcome to express your genuine thoughts and experiences. There are no right or wrong answers.

Before we begin, do you have any questions for me?

Questions about the Game (Initial Version):

1. What did you think about the game?
    a. Was it fun? Why?
    b. Was there anything difficult to understand? Why?
    c. What did you like about it? What did you not like about it?
    d. How many points did you score? How did you score these? How do you feel about it?

Questions about the Game (Further iterations):

1. What do you think about this game? Do you find it similar or different to the earlier one?
2. Which one do you like more? Why?
3. Which one was easier to understand and play??

Questions on understanding of the Game Content:

1. Can you share your understanding of the story that you heard in the game?
2. Do you think other girls like you would be able to understand this? What might they find most difficult about it?

IG Males

Interview Format – Using a semi-structured discussion guide

Thank you for taking the time to talk with me today. I would like to interview you for 1 **hour** and this will be more like a discussion.

Please note that we are not evaluating you or the program with this interview. We are hoping to learn from you, in your own words, and in your own judgment, about various aspects of the program and its influence on the young women, their families, friends and others in the community. This discussion, and any responses you provide will remain anonymous.
Before we begin, do you have any questions for me?

General Context Questions:

1. Can you please tell us a little about yourself?
    a. Your age?
    b. Religion
    c. Marital status
    d. Work
    e. Do you have a family?
2. What kinds of concerns do you have in managing the relationships you're currently engaged in?
3. Are there any risks that you feel like you're taking?
    a. Financial
    b. Health
    c. Social
    d. Legal

HIV/ Sex related campaigns

1. Are you aware of any social efforts to manage the risks of sexual behavior and the spread/ consequences of HIV (and/ or related STIs)?
    a. What is your impression of these efforts?
2. Are you aware of the Fataki campaign?
    a. What's your impression of those messages?
3. Do you feel like they are speaking to you as an actor in those stories? How so?
4. What difference have these campaigns had on your day-to-day life?
    a. On your behavior?
    b. On the behavior of your girlfriend(s)?
    c. On the behavior of the strangers you might have sex with?
5. Are you aware of Cash Transfer programs that are providing education and resources to YW to help provide opportunities for them? (if not, explain the broad details of the programs to prime the discussion)
    a. What is your impression of those campaigns?

    b. What kind of influence do you think those kinds of campaigns might have on YW?
    c. Do these kinds of efforts have an influence on you in any way?
        i. Might they change the relationships you have with women?
6. What if YW no longer needed to rely on people like yourself for resources?
    a. What differences might you see in your day-to-day life?
    b. Might that change the nature of your relationship with your girlfriend(s)?
    c. Might that change the nature of your interaction with strangers you proposition for sex/ relationships?

Peer Males
Interview Format – Using a semi-structured discussion guide

Thank you for taking the time to talk with me today. I would like to interview you for 1 **hour** and this will be more like a discussion.

Please note that we are not evaluating you or the program with this interview. We are hoping to learn from you, in your own words, and in your own judgment, about various aspects of the program and its influence on the young women, their families, friends and others in the community. This discussion, and any responses you provide will remain anonymous.
Before we begin, do you have any questions for me?

1. Tell me a little about yourself
    a. Age
    b. Level of education
    c. Religion
    d. Are you in school?
        i. Where do you go to school?
        ii. What are you hoping to do once you get out of school?
    e. Are you working?
        i. Where?

2. I'd like to talk about relationships between the boys and girls in your school and neighborhood.
    a. Do you have a girlfriend? Or girlfriends? (context and history of relationships)
        i. Is she in the same year as you?
        ii. How long have you been together?
        iii. Are you two serious? Do you have plans for a family, or is it not so certain?
3. I'm curious to learn what you've seen with women your age regarding dating habits, relationship habits and sexual behavior.

a. Do you see any of your female friends in relationships with men that are significantly older than them?
    i. What do you think about those kinds of relationships?
    ii. Why do you think the girls are involved with those men?
        1. Are they more interested in financial and resource security?
        2. What about social status reasons?
            a. What are people saying about these kinds of relationships?
    iii. Are these kinds of relationships something that the girls would choose on their own, or do you think they're kind of forced into it – either by the men, or by their overall situation in life?
        1. Do you think if the girls had a choice, would they still engage in those kinds of relationships?
    iv. Do you think that girls have any other options in life except to find a man? Either to start a family, or to help take care of them?
        1. Do girls feel like they can be independent if they want to be?
        2. Do you think they want to be?

4. Do you know of any girls, who are about your age, who are working or who have started businesses? (Tell me more)
    b. Is earning and managing money something that girls your age are concerned with?

5. Are you aware of any social campaigns that deal with sexual risks and/ or sexual behavior?
    c. Can you tell me a bit about the ones that you're familiar with?
    d. Have you heard of a Cash Transfer program?
        i. (if not, explain the general details)
        ii. What do you know/ have you heard about it?
        iii. Do you know anyone who's participating in it?
            1. Have you talked with them about it?
            2. What have they shared with you?
        iv. What is your impression of that kind of program?
        v. What do you think the girls are going to do with the money?
        vi. Do you think that it is going to make any difference on their relationship choices?
            1. How/ Why?

ETHNOLAB DISCUSSION GUIDE

finalmile.

INTRO SCENARIO 1

Testing Goals/ Questions:

None - warm up game

Scenario:

X is at home on Sunday afternoon and is bored. She have three options of how to have fun for the rest of the day.

a) She can spend time with her siblings at home

b) She can meet her friends in the market place

c) She can go meet her aunt who lives in a nearby village

Which one is she most likely to do on a Sunday?

INTRO SCENARIOS 2

Scenario:

There is an outbreak of Malaria in X's neighborhood. She is worried about her family falling sick and thinks of a few ways to know more about the disease.

a) She speaks to her neighbor who had malaria last year and knows the cost of the medication.

b) She speaks to a local pharmacist who gives her a pamphlet which has the basic information about the disease.

c) She speaks to her mother who tells her to use a mosquito net.

Which person makes her worries disappear?

Testing Goals/ Questions:

None - warm up game

RESEARCH SCENARIOS: AGYW

INTERVENTIONS LEVERS FOR AGYW

COPING & SELF WORTH

Testing Goals/ Questions:

Role modeling: What is the most inspiring/ effective role model for internalizing goal of self-reliance?

Principles/Hypotheses:

A. Positive deviances - highly successful FEAs

B. Relatable personal journey - at-risk to FEA journey

C. Successful peer from community

Scenario:

X, Y and Z are adolescent girls who have recently joined the Mpengo Pesa program in their respective areas. The three girls are not sure if starting a business is a good option for them. As part of the program, their groups meet some business women who share their own success stories with them.

X's group meets a woman who runs a very successful and large business, which she started herself with very little funds.

Y's group meets a woman who had to drop out of school due to pregnancy, but became self-reliant by starting her own business.

Z's group meets a woman from their own area who started her own business and became self-reliant.

Who among the 3 girls is likely to be most inspired to start her own business?

Discussion Guide:

Do you have a role model or someone you want to be like? What about them inspires you? When and how did you start to look up to them?

Do you know someone who reminded you of any of the options? What is similar between the two?

Do you look up to anyone in your classes or support group? Why?

INTERVENTIONS LEVERS FOR AGYW

COPING & SELF WORTH

Testing Goals/ Questions:

Building self-worth & coping: How might we increase the self-worth for AGYW to realize her goals?

Principles/Hypotheses:

A. Role model

B. Constructive Peer Network

C. Identifying skills/talents

Scenario:

X is an an adolescent who participated in the Mpengo Pesa program. She recently started a business of selling soap but is struggling to make it successful. The program has the following options to help her overcome the struggle:

a) Provide her a mentor that has been running a business for 10 years,

b) Connect with a support group of girls of her age who also wish to start their own business

c) Provide her business and cash management training.

Which option may be the best way for help her overcome her challenges?

Discussion Guide:

Do you run a business or plan to start a business? Do you know other girls of your age who do? What inspired you/this girl - any person or event?

Do you discuss anything about work and money with your friends? What do you discuss? Have they helped you in any way?

Do you have a skill which will help you in your work? How did you get it? Did someone help?

INTERVENTIONS LEVERS FOR AGYW

COPING & SELF WORTH

Testing Goals/ Questions:

Barriers: What are the critical barriers to overall empowerment for women in general and in starting a business in particular? How might they differ for different women?

Principles/Hypotheses:

A. Resistance from family/partner

B. Avoidance/low confidence

C. Insufficient funds

Scenario:

X is an adolescent girl who has recently joined the Mpengo Pesa program. X is planning to start a business of selling soaps. However, she is one of the following issues that is preventing her to do that:

a) X's family and partner is opposed to women working;

b) X does not feel confident about making decisions herself, so she depends on her parents' decisions regarding money;

c) X does not find the money she receives from the Mpengo Pesa program sufficient to start a business

Which of the 3 reason is likely to be the reason that is preventing X to start her own business?

Discussion Guide:

Do you think your family and partner are ok with you starting a business? What are the other barriers that you see in the community?

Which risks and problems should a girl be prepared for when starting a business?

Do some girls/young women come together and pool in their resources to start a business?

INTERVENTIONS LEVERS FOR AGYW

COPING & SELF WORTH

Testing Goals/ Questions:

Perceived worth for CT: How does AGYW evaluate the value of the quantum of CT? How might this differ for different women?

Principles/Hypotheses:

A. Start a business

B. Satisfies Basic needs

C. Satisfies luxurious wants

Scenario:

X, Y and Z are five girls who have joined the Mpengo Pesa program in their respective areas. They receive money through a mobile phone. Through the next 3 months each of them use it for different purposes.

X uses it to buy goods to start her own business.

Y uses it to buy basic household supplies like oil and food, but more quantity or better quality than earlier.

Z uses it to buy personal supplies like make-up and clothes.

Which girl uses the CT money like the rest of her peers- X, Y or Z?

Discussion Guide:

What did you do with the money you received? How do most girls spend it?

Which of your goals or wishes does the money you receive help you achieve?

Are some girls using it to start a business? What kind of business?

INTERVENTIONS LEVERS FOR AGYW

GOAL SETTING

Testing Goals/ Questions:

Long term goals to promote saving: What motivates saving behaviors in women?

Principles/Hypotheses:

A. Family goals - health, education

B. Personal goals - capital, skilling

C. Emergency funds

Scenario:

X, Y and Z are girls who are discussing the importance of savings. In this discussion, the three of them consider different benefits of savings.

X says that savings could help them address family responsibilities like children's nutrition, health and education

Y says that savings could help girls gain new skills like tailoring, making soap, etc. to start their own business

Z says that savings could help the them address emergency situations in the future and cope with difficult times

Which girl is most likely to save part of her income- X, Y or Z?

Discussion Guide:

Did you save any money? Where do you save? How much do you have now?

Why do you want to save? Do you think other girls are saving? What might be their reasons?

What are the difficulties that you and other girls like you may face in saving money?

INTERVENTIONS LEVERS FOR AGYW

GOAL SETTING

Testing Goals/ Questions:

Conditions enabling exit from IG/TS/MP (inter-generational, Transactional Sex, Multiple Partner): What is the intrinsic motivation that is driving AGYW to exit risky relationships?

Principles/Hypotheses:

A. Risk perception

B. Efficacy/Resource access

C. Social proof and norms

Scenario:

X, Y and Z are girls who are in multiple relationships and they are considering leaving whether they should limit their relationships to one partner.

X feels that she faces risk of HIV infection because her partners may have other partners who are infected

Y has started earning her own income for selling vegetables, with which she can meet her basic needs

Z spends time with a group of friends who all have single partners in long-term relationships

Who is most likely to leave her relationships with multiple partners- X, Y or Z?

Discussion Guide:

What are the reasons for girls having multiple partners in your community? What are the challenges of managing multiple relationships?

What might be the reasons for girls to switch from multiple partners to only one? Are there any challenges in this decision? Do you speak to others about this decision?

INTERVENTIONS LEVERS FOR AGYW

GOAL SETTING

Testing Goals/ Questions:

Motivations for financial empowerment: What intrinsic value might women see in financial empowerment? How might they differ for different women?

Principles/Hypotheses:

A. More power and status in family/community, more control

B. Personal growth and satisfaction

C. Prudence, aspiration, money for future expenses

Scenario:

X is a girl participating in the Mpengo Pesa program, who is considering starting her own business with the money she has received in the program. She is thinking what might be the benefits of this action.

A. Having own income would give her ability to make her own decisions and more power and status in her relationship and in the community

B. Running a business would teach her new skills and help her be a part of a group of self-reliant girls.

C. If she starts earning, she can hope for a better future for herself and her family

Which benefit is she likely to favor in her decision?

Discussion Guide:

How do you think running your own business will help you?What are the barriers that might prevent you from reaching these goals?

Which kind of business would you like to learn about?

How do you think your family and partner would react to you starting a new business?

INTERVENTIONS LEVERS FOR AGYW

GOAL SETTING

Testing Goals/ Questions:

Structure of dispersal: Does the structure of CT dispersal impact AGYW's expectation of using the money?

Principles/Hypotheses:

A. Mental accounts

B. Staggered

C. Gradated

Scenario:

X, Y and Z are receiving Mpengo Pesa, but there are some differences in how they receive the money. All of them receive the same amount - 70k after every 3 months.

X receives money under 3 accounts at the same time, once in 3 months - 20k for personal expenses, 20k for savings and 30k for starting business. She can spend money as she likes.

Y receives 10k 7 times during the 3 months, which adds to total of 70k, instead of all at once.

Z receives 3 installments during the 3 months that add to total of 70k, instead of all at once - 10k in first month, 20k in second month and 40k in third month.

Who is likely to be less dependent on others for money?

Discussion Guide:

How long did the money that you received lasted?

Did you plan how you would spend it? Did you do everything as planned or were there any changes?

INTERVENTIONS LEVERS FOR AGYW

EFFICACY & ABILITY

Testing Goals/ Questions:

Financial Literacy - How might we encourage women to take loans?

Principles/Hypotheses:

A Confidence of paying back (self control, self-efficacy)

B. Trust in the group and treasurer

C. Risk-appetite, optimism

Scenario:

X, Y and Z are girls who are considering taking loans from their support groups for starting their own business.

X is confident that she will pay back the loan even if she has to compromise on her own needs.

Y trusts the girls in her group, especially the people in-charge of managing the money.

Z is optimistic that the business she starts with the loan will be successful, and she will have enough money to pay back.

Who is most likely to take the loan?

Discussion Guide:

Have you ever borrowed money from someone? From whom? When did you pay back?

What are the reasons for borrowing in your community? What discourages you to take a loan?

Do you plan to borrow from the support group? For what reason?

INTERVENTIONS LEVERS FOR AGYW

EFFICACY & ABILITY

Testing Goals/ Questions:

Barriers obstructing the efficacy / ability to empowerment: What are the barriers that are still obstructing AGYW's ability towards empowerment?

Principles/Hypotheses:

A. Avoiding immediate costs for gain in future - avoid confrontation/conflict with partner

B. Avoid effort & risk, status quo - keep both resource channels open - partner & CT

C. CT amount not sufficient to meet current needs, therefore unable to spare for starting business

Scenarios:

X, Y and Z are participants of the Mpengo Pesa program. In last week's support group, the teacher taught the girls to make soaps. At the end of the class, they are encouraged to start thinking about how they might create a business out of selling soaps. While listening to the teacher , they begin to think of the problems that might not allow them to start the business?

X knows that even though starting a business will help her in the future, it will create a conflict in her relationship with partner today

Y wants to avoid the risk and effort to start a business and is currently satisfied with the lifestyle and resources that come with the Mpengo Pesa amount and her partner.

Z thinks that there are too many expenses in the present that there is isn't enough money to spare to start a business

Whose problem is most relevant to prevent the girls to start the business of selling soaps?

Discussion Guide:

How much money do you need for your personal expenses in a month? Does your partner give you any money?

Do you think starting your own business is a good idea? What might be the challenges in starting own business?

Do you take advice on these matters from anyone?

INTERVENTIONS LEVERS FOR AGYW

EFFICACY & ABILITY

Testing Goals/ Questions:

Anticipated Barriers: What barriers might prevent activation of workshop knowledge (e.g. condom negotiation)

Principles/Hypotheses:

A. Fear of consequences

B. Low Retention

C. Low Relevance at home

Scenario:

X, Y and Z have recently been introduced to topics around condom use, family planning, and STIs in their support groups. They felt encouraged and empowered in the workshop setting. However, they approach the teacher after the class. Each of them anticipates barriers to activation of the knowledge.

X is afraid that her partner might get aggressive/ abusive if she approaches him about condom usage

Y is concerned that while these topics are relevant, she might have difficulty retaining the information she just received

Z is unsure if these subjects will be relevant since she is a long term relationship with one partner.

Which girl's concern is most relevant here?

Discussion Guide:

What are the things that you were taught in the classes of your support group? Which topic did you find most interesting?

What is the biggest challenge with the classes? Do you often forget what you are taught? Are some topics difficult to understand? Do you think the classes are not useful for you? What is the reason?

INTERVENTIONS LEVERS FOR AGYW

EFFICACY & ABILITY

Testing Goals/ Questions:

Low Risk Perception: What might be the reason for low / acceptable risk when it comes to IG / TS relationships?

Principles/Hypotheses:

A. Self-other discrepancy

B. Present bias/tunneling

C. Desensitization/Normative

Scenario:

X, Y and Z participate in a support group where they are taught about the risks of HIV infection and steps for prevention. All three have multiple sexual partners, but the girls are not convinced about the risk.

X believes that she makes good decisions regarding relationships, so she doesn't need to worry. Other girls face risk of HIV as they make bad decisions.

Y really enjoys spending time with her partners, and she trusts that there is no risk involved in her relationships. She doesn't like to worry about it.

Z feels that HIV is so common in the community, that her behavior does not make any difference. Even with one partner, there is a risk of infection, so nothing wrong with multiple partners.

Who is most likely to forget about the lesson and continue multiple relationships?

Discussion Guide:

Which kind of relationship should a girl avoid - multiple partners at once, a partner who is much older than her, or relationship with expectation of money in return? What is the reason for this?

Which kind of girls face the highest risk of HIV infection? Do you have more or less risk than an average girl in your community of contracting HIV? Why?

How might you be able to reduce the risk you face of contracting HIV?

INTERVENTIONS LEVERS FOR AGYW

SOCIAL EMBEDDING

Testing Goals/ Questions:

Social Impact: What is the social impact of CT on AGYW?

Principles/Hypotheses:

A. Increased respect and status

B. Becoming a target of envy and stereotypes due to resource access

C. Perceived as sexual deviant/rogue

Scenario:

X is a girl participating in a Mpengo Pesa program. Her neighbors and friends are also aware of her participation and she is thinking about their perception of her.

She feels that people around her give her more respect

She is worried that people around her may be jealous of her money

She feels that people might think she has been having sex to get money in return

What is she likely to think about the people's perception in her community?

Discussion Guide:

What do the people of your community think about the Mpengo Pesa program?

Do you face any challenges because of having access to this money? Have any other girls faced any problems due to the money?

INTERVENTIONS LEVERS FOR AGYW

SOCIAL EMBEDDING

Testing Goals/ Questions:

Impact of CT rollback: Do the women perceive that CT has changed status quo? What might happen if it is withdrawn?

Principles/Hypotheses:

A. Back to BAU

B. Group Self-reliance

C. Backlash/disruption

Scenario:

X is participating in a workshop, where the teacher informs her that the upcoming Mpengo Pesa will be the last amount that she will be receiving. She had grown accustomed to receiving this amount for her new business.

She thinks it is best pursue an additional boy friend to substitute the cash transfer to meet her needs.

She thinks it is best to continue to stay connected with her peer group from Mpengo Pesa program and build her soap business.

She thinks that she will now have to worry about new issues at home since her partner was anyways not in favor of the Mpengo Pesa program.

How do you think she is likely to react?

Discussion Guide:

How long do you think you will receive money from the Mpengo Pesa program? How long would you like this to continue?

Do you think about what might happen when you stop getting money from the program? What kind of problems might you face when that happens?

INTERVENTIONS LEVERS FOR AGYW

SOCIAL EMBEDDING

Testing Goals/ Questions:

Framing to promote advocacy of empowerment: How do we make CT participants encourage other women towards empowerment?

Principles/Hypotheses:

A. Helping fellow women with similar difficulties

B. Less risky sexual behaviors in community

C. Becoming a role-model

Scenario:

X, Y and Z are given the task of informing the girls in their community about the use of condom to prevent HIV infection.

X wants to help fellow women in her community who face similar sexual risks such as acquiring HIV

Y wants to live in a society where there are less people with HIV

Z wants to be seen as a role-model by other girls in her community, and wants to guide them towards better decisions.

Who is likely to perform the task with most dedication?

Discussion Guide:

Who do you talk to about the topics that the teacher discusses in the support groups? Which topic would you like to share with other girls?

Do you think girls who are not a part of the program should also learn about these topics? What is the reason?

According to you, what do you think motivates a person to be cautious and use condoms/ negotiate condom usage?

RESEARCH SCENARIOS: FEW

UNDERSTANDING FEW (Financially Empowered Women)

KEY REQUISITES

Testing Goals/ Questions:

Key behavioral requisites to self-reliance: What are the components of self-efficacy for an FEA?

Principles/Hypotheses:

A. Risk appetite

B. Negotiation/people skills

C. Resilience/Coping

Scenario:

X, Y and Z are three young women who have recently started their own businesses of selling vegetables in the local market of their respective towns. All three of them hope to achieve success in their respective business, but their personalities/working styles are quite different from each other.

X finds it exciting run business where there is an opportunity to make very large profits, even if there is some possibility of loss as well.

Y is very good at conversing with people she interacts with in the market and is very persuasive in business negotiations and bargains.

Z can deal with the ups and downs of the business easily, and can recover very comfortably from difficulties she may face in the business.

Who among the 3 young women is most likely to achieve success in her business?

Discussion Guide:

What made you realize that you can run a successful business and why?

What do you think is the most important skills to run a successful business?

UNDERSTANDING FEW (Financially Empowered Women)

EXPECTATIONS

Testing Goals/ Questions:

Impact of self-reliance: How do FEAs define empowerment?

Principles/Hypotheses:

A. Self-confidence and self-worth

B. Status and reputation

C. Control and coping potential

Scenario:

X, Y and Z are three young women who run their own food stalls. The three have different views of how running their own business has affected them personally.

X feels that she has gained more confidence in her capabilities and values herself more.

Y feels that she has gained a good reputation in the community and people value her more.

Z feels that she has gained more power to make her own decisions and can deal with difficulties independently.

Which impact of running own business is more likely?

Discussion Guide:

What created a desire for you to run a business by yourself?

How did you create a vision for yourself?

UNDERSTANDING FEW (Financially Empowered Women)

ENABLERS

Testing Goals/ Questions:

Conditions enabling success in business: How did FEA evolve to become self-reliant?

Principles/Hypotheses:

A. Supportive family/relationship

B. Trustworthy business partners

C. Role models

Scenario:

X, Y, and Z are three women successfully run food stalls. An opportunity to earn double of their monthly revenue comes their way. Before they consider accepting it, the women try and understand what is most important to ensure the success of this opportunity.

X thinks it's possible because her partner and family are supportive of her business. The more she can earn, the better it is for the family.

Y thinks it's possible because her business partners are hardworking and trustworthy. Together, they can earn more, get more experience and add to their savings for the business.

Z thinks it's possible because her neighbor who is her role model runs a food stall and is willing to guide her through this business opportunity.

Who among the three is likely to take the opportunity to earn more for their business- X, Y or Z?

Discussion Guide:

Where else might the confidence to take a decision like this come from? (experience, reputation, risk appetite)?

UNDERSTANDING FEW (Financially Empowered Women)

ENABLERS

Testing Goals/ Questions:

Behaviors enabling success in business: How did FEA evolve to become self-reliant?

Principles/Hypotheses:

A. Regular, systematic saving

B. Networking, building rapport and reputation

C. Balancing domestic responsibilities

Scenario:

X, Y and Z are three women who have recently started their own business. They have acquired new habits while running business.

X saves a fixed amount every week and puts it away for future use

Y spends time everyday networking, building rapport and gaining a good reputation in her community and among her peers

Z equally splits her time attending to her domestic responsibilities and balancing it with her businesses

Who do you think will be most successful in ruNning their business?

Discussion Guide:

Do you discuss how you save/ spend money from the CT program? Who do you discuss it with? What works/ doesn't work for you?

Do you earn a consistent amount every month? If not, how do you manage your earnings?

If you don't earn an adequate amount on a particular month, what do you do? (borrow, take from savings, reduce the regular expenditure- tradeoffs)

UNDERSTANDING FEW (Financially Empowered Women)

ENABLERS

Testing Goals/ Questions:

Aides for managing business: How might we improve AGYW's ability to manage business?

Principles/Hypotheses:

A. Business mentor

B. Connecting with potential collaborators

C. Business roadmap

Scenario:

X, Y and Z are earning women participating in support groups to learn about running a business. They receive some assistance in the workshops for improving their business skills.

X was assigned a successful business woman from her community to guide her in making difficult decisions.

Y was connected with another women who run similar businesses and are willing to collaborate.

Z was helped with creating a business plan to improve and expand her current business and grow it faster in future.

Which woman will find the workshop most helpful?

Discussion Guide:

One time learning vs. modules over the years that help track one's business.

What do you think are the biggest concerns about running a business?
